# Individuals cannot rely on COVID-19 herd immunity: Durable immunity to viral disease is limited to viruses with obligate viremic spread

**DOI:** 10.1371/journal.ppat.1009509

**Published:** 2021-04-26

**Authors:** Jonathan W. Yewdell

**Affiliations:** Cellular Biology Section, Laboratory of Viral Diseases, NIAID, Bethesda, Maryland, United States of America; University of Colorado Denver, UNITED STATES

It is often messaged that herd immunity to Severe Acute Respiratory Syndrome Coronavirus 2 (SARS-CoV2 (CoV-2)), the causative agent of Coronavirus Disease 2019 (COVID-19), will protect nonvaccinated individuals from infection. Herd immunity refers to the concept that when a sufficient fraction of individuals in a population develop immunity from infection or vaccination, viral transmission is reduced to a near negligible level. However, seasonal CoVs, which cause approximately 20% of common colds, remain endemic, even though demonstrating only limited antigenic evolution in epitopes targeted by neutralizing antibodies [1,2]. Although there are only 4 circulating seasonal CoVs, infections frequently recur, even yearly, likely related to waning antibody levels [3]. Human challenge studies established that seasonal CoV reinfection with the identical strain can occur within a year after initial exposure, though typically with reduced shedding and milder symptoms [4]. Reinfection also appears to occur following mild COVID-19 cases, where the serum neutralizing antibody half-life is only approximately 5 weeks [5].

If immunity to SARS-CoV-2 and seasonal CoVs are similar, COVID-19 herd immunity is a pipe dream, even more so given the relatively rapid selection of mutants with amino acid substitutions in the spike protein that reduce the efficiency of serum antibody neutralization [6]. Absent effective herd immunity, over the next few years, individuals can choose whether their first exposure to SARS-CoV-2 immunogens occurs via vaccination or infection. With the age-related increase in COVID 19 severity, it is critical that individuals be vaccinated sooner rather than later.

Coronaviruses are hardly unique in their ability to reinfect humans. Infection with none of the common endemic human respiratory viruses consistently induces durable immunity (Table 1). Although influenza A and B viruses are notorious in this regard, they are, in a sense, less adept than the other respiratory viruses, which reinfect individuals without resorting to significant antigenic variation. Similarly, many viruses that infect the gastrointestinal (GI) tract can infect vaccinated or previously infected individuals, most likely due to waning immunity (Table 1). Unlike respiratory viruses, however, several GI viruses are well controlled by infection or vaccination, including poliovirus, now on the brink of vaccine-induced extinction.

**Table 1:** Characteristics of infection and immunity for human viruses.

Virus	Initial Infection Site	Lymph/Blood Dissemination for Disease/Transmission	Durable Immunity Infection	Durable Immunity Vaccination
Corona	Airway	No	No	N/A
Influenza A, B	Airway	No	No	No
Metapneumonia	Airway	No	No	No
Parainfluenza 1–3	Airway	No	No	N/A
Respiratory Syncytial	Airway	No	No	N/A
Rhino	Airway	No	No	N/A
Ebola	Airway	Yes	Yes	Yes
Measles	Airway	Yes	Yes	Yes
Mumps	Airway	Yes	Yes	Yes
Parvovirus	Airway	Yes	Yes	N/A
Rubella	Airway	Yes	Yes	Yes
Varicella	Airway	Yes	Yes	Yes
Variola	Airway	Yes	Yes	Yes
Noro	Gastrointestinal	No	No	
Rota	Gastrointestinal	No	No	No
Hepatitis A	Gastrointestinal	Yes	Yes	Yes
Polio	Gastrointestinal	Yes	Yes	Yes
Dengue fever	Blood	Yes	Yes	Yes
Hepatitis B	Blood	Yes	Yes	Yes
Yellow Fever	Blood	Yes	Yes	Yes

Poliovirus vaccination provides insight into the nature of protective antiviral immunity. Intramuscular immunization with inactivated virus prevents paralytic disease but not GI infection, with repeat vaccination necessary to reduce shedding of infectious virus. Similarly, even natural respiratory infections with measles or variola (smallpox) viruses, famous for inducing life-long immunity to disease, do not prevent respiratory reinfection, which though asymptomatic and nontransmissible, can be detected by increased antiviral antibody titers [7,8].

What polio-, variola, and measles virus share is dissemination from the initial infection site via lymph and (secondarily) blood as an obligate step in pathogenesis or transmission. Virus-programmed interorgan dissemination occurs in stages over days, as the virus productively infects one organ and proceeds to the next via lymph and blood [9]. Table 1 [10] summarizes the protection conferred by natural infection/vaccination for 20 common human viral pathogens. Blood/lymph-based dissemination or tropism is clearly implicated as the critical vulnerability of viruses to infection/vaccination-induced immunity.

By contrast, immune memory routinely fails to control viral reinfections on mucosae. At first glance, this might be surprising, as 90% of synthesized antibodies (the vast majority being immunoglobulin A (IgA)) are delivered to mucosae. Yet selective IgA deficiency, the most common inherited immune deficiency in Caucasians, has modest effects on susceptibility to mucosal viruses [11]. This suggests that evolution may have focused antiviral immunity on preventing virus dissemination via body fluids rather than blocking mucosal infection.

Antiviral antibodies are present at relatively high concentrations in blood and lymph, where they can function directly by neutralizing viral infectivity and indirectly via Fc-mediated viral clearance by phagocytic cells and natural killer (NK) cell killing. Phagocytic clearance is particularly effective in the lymph nodes, which evolved in part to eradicate particulate pathogens. Critically, limiting virus dissemination in body fluids blocks virus pathogenicity in end-organs and prevents end-organ participation in viral transmission.

While vaccines are unlikely to provide long-lasting sterilizing immunity to CoV-2, they should durably reduce viral replication in the upper airway and prevent, or at least attenuate, pulmonary disease and virus transmission. Further, vaccines should be highly effective at preventing pathogenicity resulting from viremia, which includes intestinal and renal dysfunction, systemic hypercoagulation, and endothelial inflammation. Although the degree to which the Moderna and BioNTech mRNA vaccines owe their remarkable protectiveness is due to humoral versus cellular immunity remains to be determined, the extremely robust antibody response they elicit is consistent with a major role for neutralizing antibodies in disease protection [12,13].

Why does the immune system have difficulty controlling mucosal viral infections? In part, this may be due to the wiliness of viruses, whose survival requires staying one step ahead of host immunity. As the skin provides an extremely effective physical barrier to viruses, typically only breached by insect bites, mucosae are the primary site of viral transmission. Mucosae are replete with immune tissues that possess the bulk of antibody-secreting B cells. Most mucosal antibodies function to control the commensal cellular microbiome, which requires constant shepherding lest it overwhelm the host. Enteric viruses can bind to bacteria to enhance attachment and infection of epithelial cells [14,15]. Could this also shield them from antibodies?

Several other possible factors contribute to the limited human mucosal immunity to virus reinfection. First, until approximately 10,000 years ago, humans evolved in isolated small populations, and viral infections were likely mostly limited to viruses capable of vertical transmission (herpesviruses, papillomaviruses) and arboviruses (i.e., transmitted to the blood by insects). Under these conditions, there may have been little selection pressure to deal with horizontally transmitted viruses that infect mucosae. Comparing mucosal immunity between vertebrates that evolved in small versus large population clusters may reveal immune mechanisms that more effectively control mucosal infections, leading to insights for improved vaccination and treatment.

Second, the immune system may have evolved to permit viral mucosal infections. If mucosal infection was a significant threat to host reproduction, wouldn’t innate antiviral mechanisms (e.g., type I interferon (IFN)-stimulated genes) be employed constitutively in mucosae rather than conditionally? Although antiviral cytokines exert potent effects on the nervous system that would impact evolutionary fitness if present chronically (fever, malaise, etc.), it seems likely that evolution could have crafted a solution to localize innate antiviral effects. Could virus-mediated horizontal gene transfer provide sufficient evolutionary advantages to temper antiviral immunity [16]? Could limited, but repeated, mucosal infections play a role in maintaining basal immunity to more serious pathogens, as described for chronic herpesvirus infections [17]?

With better understanding of immunity, it may be possible to develop vaccines that provide far more robust immunity than natural infections and overcome mucosal immunity’s limitations. An example is the effectiveness of the *Salmonella typhi* Vi capsular polysaccharide vaccine, which targets a bacterial component nonimmunogenic in natural infections. Indeed, it is possible that mRNA immunization provides more durable protection than SARS-CoV-2 infection, given that vaccination induces higher antibody titers than infection [18]. The cost of developing effective vaccines to the many present (and future) mucosal viruses that plague humanity is a pittance relative to the havoc caused by our ignorance. COVID 19, as terrible as it has been, should be a wake-up call: Far more dangerous viruses lurk in nature.
